# 322例非小细胞肺癌骨转移临床特点及治疗的回顾性分析

**DOI:** 10.3779/j.issn.1009-3419.2014.09.03

**Published:** 2014-09-20

**Authors:** 庆志 郭, 梅娜 吴, 彤同 安, 军 赵, 建春 段, 志杰 王, 书航 王, 洁 王

**Affiliations:** 100142 北京，北京大学肿瘤医院暨北京市肿瘤防治研究所胸部肿瘤内科，恶性肿瘤发病机制及转化研究教育部重点实验室 Key Laboratory of Carcinogenesis and Translational Research (Ministry of Education), Department of Toracic Oncology, Peking University School of Oncology, Beijing Cancer Hospital and Institute, Beijing 100142, China

**Keywords:** 肺肿瘤, 骨转移, ECT, CT, MRI, X线, Lung neoplasms, Bone metastases, ECT, CT, MRI, X-ray

## Abstract

**背景与目的:**

骨是非小细胞肺癌最常见转移部位之一，可引起疼痛病理性骨折等，严重影响患者生活质量。本研究探讨非小细胞肺癌骨转移的临床特点及预后因素。

**方法:**

回顾性分析我科近5年收治的600例非小细胞肺癌患者，单光子计算机断层扫描（emission computed tomography, ECT）作为筛查方法，计算机断层扫描（computed tomography, CT）/磁共振成像（magnetic resonance imaging, MRI）/X线或病理学诊断作为骨转移的确诊方法。

**结果:**

肺腺癌发生骨转移的比率最高，脊柱、骨盆、股骨等为骨转移高发部位。ECT显示3个浓聚灶及以上者，行CT/MRI/X线可以证实骨转移，确诊骨转移的几率远远高于ECT显示1个-2个浓聚灶的患者[80.6%（203/252）*vs* 50.79%（32/63）, *P* < 0.001]。出现骨转移后未发生骨相关事件（skeletal related events, SRE）的患者生存期长于发生SRE者，1年生存率和中位生存期分别为44.75%、14.74个月 *vs* 36.17%、12.25个月（*P*=0.022）。经*Cox*多因素分析，病理组织学诊断为非腺癌、骨转移病灶数少于3个，单纯骨转移为有益于生存期的预后因素。

**结论:**

ECT异常浓聚灶的数目与骨转移影像学的确诊有关。病理组织学非腺癌、骨转移病灶数少于3个、单纯骨转移是非小细胞肺癌骨转移的独立预后因素。

## Abstract (en)

**Background and objective:**

Bone metastases are the most common metastases of the non-small cell lung cancer (NSCLC). It can lead to bone ache and pathology fracture, deteriorate the quality of life.

**Methods:**

We retrospectively investigated the characteristics, diagnosis and prognosis factors of bone metastases in NSCLC. All of the 600 patients are from department of thoracic oncology in recently 5 years. Emission computed tomography (ECT) was used to screen the bone metastases and the diagnosis of bone metastases was confrmed by computed tomography (CT)/magnetic resonance imaging (MRI)/X-ray or pathology.

**Results:**

Among the total 322 bone metastases patients, subtype of adenocarcinoma had the most opportunity to occur bone metastases, and we found that vertebrae, pelvis and femora *et al* were the most frequently involved metastases sites. Patients who had more than 3 high ^18^F-FDG uptake sites of ECT, could be confrmed bone metastases by CT/MRI/X-ray than those with 1-2 high ^18^F-FDG uptake sites [80.6% (203/252) *vs* 50.79% (32/63), *P* < 0.001]. The patients with bone metastases who had non-SRE had longer survival than that of SRE [1-yr survival 44.75% (non-SRE) *vs* 36.17% (SRE); median survival 14.74 mo (non-SRE) *vs* 12.25 mo (SRE)]. Multivariables analysis showed the pathology were non-adnocarcinoma, bone metastases less than 3 sites and bone metastases without other organs metastases would have good prognosis.

**Conclusion:**

Tere were relations between the numbers of abnormal dense sites of ECT and the diagnosis of skeletal metastases, non-adnocarcinoma bone metastases less than 3 sites and bone metastases without other organs metastases were the independent prognosis factors..

肺癌是我国发病率和死亡率最高的恶性肿瘤，2012年中国肿瘤登记年报显示：肺癌发病率及死亡率分别为53.57/10万和45.57/10万^[1]^，且发病隐秘，不易早期诊断，确诊时50%为晚期，骨是非小细胞肺癌(nonsmall cell lung cancer, NSCLC)主要的血循转移部位之一。骨转移后的骨相关事件(skeletal related events, SRE)严重影响患者生活质量和生存期^[2, 3]^。骨转移早期诊断对于预防和治疗SRE具有重要的意义。骨发射单光子计算机断层扫描(emission computed tomography, ECT)是最常用的筛查骨转移的方法，但是其假阳性率较高。X线特异性高、操作简单、能基本显示骨质密度变化且费用低廉，可对其他影像检查发现的骨质异常进行进一步确认，但其敏感性低。计算机断层扫描(computed tomography, CT)可以显示骨骼的细微结构，为ECT阳性患者的确诊性检查，以明确是否有骨破坏、并了解破坏程度。磁共振成像(magnetic resonance imaging, MRI)诊断骨转移的敏感性和特异性均高，显示骨髓和软组织解剖清晰，诊断脊柱神经压迫症状时有优势。本研究回顾性分析了我科连续收治的600例NSCLC患者，总结肺癌骨转移的规律及诊断治疗经验。

## 1 材料及方法

### 1.1 研究对象

以北京大学临床肿瘤学院胸部肿瘤内科2006年1月-2011年12月连续收治的600例NSCLC患者为研究对象。入选标准：①病理或细胞学检查确诊为NSCLC；②肿瘤-淋巴结-转移(tumor-node-metastasis, TNM)分期为Ⅳ期；③美国东部肿瘤协作组(Eastern Cooperative Oncology Group, ECOG) ≤2；④完成全面分期检查(胸部CT、ECT、腹部B超、脑核磁或CT等)。排除标准：①双重癌或多重癌；②ECOG≥3；③无病理诊断；④未完成分期检查。患者的一般特征见表 1。

**1:** 600例NSCLC患者临床特点 Clinical characteristic of 600 patients with NSCLC

		*n* (No. of bone metastases percent)	*X*^2^ value	*P* value
Gender			0.640	0.461
	Male	310 (162, 52.26%)		
	Female	290 (160, 55.17%)		
Age (yr)			0.060	0.835
	≥70	113 (55, 48.67%)		
	﹤70	487 (267, 54.83%)		
Histology			1.616	0.806
	ADC	310 (168, 54.19%)		
	SQC	197 (104, 52.79%)		
	BAC	20 (11, 55.00%)		
	LCC	8 (6, 75.00%)		
	Others	65 (33, 50.77%)		
Cell differentiation			3.043	0.109
	Poor-moderate	548 (301, 54.93%)		
	Well	52(21, 40.38%)		
NSCLC: non-small cell lung cancer; ADC: adenocarcinoma; LCC: large cell carcinoma; SQC: squamous cell carcinoma; BAC: bronchioloalveolar carcinoma.				

### 1.2 方法

600例患者均在治疗前行ECT，对显示浓聚部位进行CT/MRI/X线检查，部分患者行局部组织穿刺活检获得病理诊断。骨转移的确诊标准为^[4]^：①临床或病理诊断肺癌，骨病变活检符合肺癌转移；②肺癌病理诊断明确，具有典型的骨转移影像学表现。排除标准：CT或MRI未见骨质破坏且随访6个月仍未见骨质破坏。确诊骨转移的患者定期行实验室监测血钙等，监测SRE的发生时间(包括高钙血症、严重骨痛需要放疗、压迫脊髓、病理性骨折等)，予双磷酸盐治疗(帕米磷酸二钠或唑来磷酸)，同时对症止痛治疗。全身治疗以含铂双药化疗为主。疗效评价以实体瘤疗效评价标准(Response Evaluation Criteria in Solid Tumors, RECIST)为准，生存期以治疗首日至死亡日期为准，以月计。

### 1.3 统计学方法

数据采用SPSS 16.0统计软件包进行处理，定性数据以百分比表示，计数资料采用*χ*^2^检验，*Kaplan-Merier*法绘制生存曲线，*Log-rank*法进行差异分析，*P* < 0.05为差异具有统计学意义。

## 2 结果

### 2.1 ECT与CT/MRI/X线结果的比较

#### 2.1.1 ECT的敏感性与特异性

600例患者中有442例ECT阳性，确诊骨转移322例，其中315例为ECT阳性同时CT/MRI/X线发现明确骨质破坏而确诊，7例ECT阴性患者因CT/MRI/X线发现骨质破坏而确诊，127例ECT阳性患者随访6个月仍未发现骨质破坏及转移症状而除外骨转移。ECT敏感性为97.83%，特异性为54.32%，准确性为77.66%，漏诊率为22.33%，假阳性率为28.73%，假阴性率为4.43%。见表 2。

**2:** ECT的敏感性及特异性 Sensitivity and specificity of ECT

	ECT positive	ECT negative	Total
Bone metastasis	315	7	322
Not bone metastasis	127	151	278
Total	442	158	600
Sensitivity: 315/(315+7)=97.83%; Specificity: 151/(127+151)=54.32%; ECT: emission computed tomograph.			

#### 2.1.2 ECT浓聚灶数目与与影像学诊断关系

ECT显示1个-2个浓聚灶和3个以上病例数分别是63例和252例，通过影像学证实率分别是50.79%(32/63)和80.56% (203/252)(*χ*^2^=23.562, *P* < 0.001)，两组有统计学差异。见表 3。

**3:** ECT阳性浓聚灶数与不同诊断方法对骨转移确诊的比较 The relationship of positive sites of ECT and bone metastases diagnosis with different methods

Numbers of positive sites	*n*	Bone damage	No bone damage	CT (+)	MRI (+)	X-ray (+)	Confirm (percent)
1-2 positive sites^*^	63	32	31	20	12	0	50.79% (32/63)
≥3 positive sites^*^	252	203	49	104	87	12	80.56% (203/252)
Total	315	235	80	124	99	12	
^*^χ^2^=23.562, *P* < 0.001; CT: computed tomography; MRI: magnetic resonance imaging.							

### 2.2 NSCLC骨转移的临床特点

2例骨转移患者中症状的有无，是否多脏器转移的例数统计学无明显差异。骨转移部位(承重骨转移、扁骨转移)，骨转移的类型(溶骨性骨转移、成骨性骨转移、混合型转移)例数上有统计学差异。脊柱为NSCLC骨转移最常见的部位，其次为骨盆、股骨、肋骨、胸骨。见表 4。

**4:** NSCLC骨转移的特点 The characteristic of bone metastases in NSCLC

Item		Percent & Number	*U* value	*P* value
Syndrome of bone metastases				> 0.05
	With syndrome	60.56% (195/322)	0.53	
	Non syndrome	39.44% (127/322)		
Sites of bone metastases			13.56	﹤0.01
	Bearing bone metastasis	65.53% (211/322)		
	Flat bone metastasis	34.47% (111/322)	
Multi-organs metastases			1.34	> 0.05
	Bone metastases only	34.16% (110/322)		
	Bone metastases with other organs	65.84% (212/322)		
Type of bone damage			6.67	﹤0.05
	Osteolysis metastases	87.58% (282/322)		
	Osteogenesis metastases	2.48% (8/322)		
	Mixing type metastases	7.76% (25/322)		
	Others	2.17% (7/322)		
Sites of bone metastases			2.34	> 0.05
	Spinal column	54.35% (175/322)		
	Pelvis	34.78% (112/322)		
	Femora	25.16% (81/322)		
	Rib	13.35% (43/322)		
	Sternum	12.42% (40/322)		
	Others	17.70% (57/322)		

### 2.3 SRE的发生率

208例患者出现了SRE，严重骨痛而放疗(188例，90.38%)为最常见的SRE，高钙血症发生率最低(4例，1.92%)。见表 5。(骨痛需要放疗的SRE定义：①非承重骨的骨转移，伴骨痛(VAS≥4分)，经中度止痛药无效而接受放疗属于SRE；②承重骨骨转移，伴疼痛(VAS≥4分)，接受放疗属于SRE。

**5:** NSCLC骨转移SRE发生情况表 SRE in NSCLC with bone metastases

Type of SRE	Percent & Number
Pathologic fractures	2.40% (5/208)
Radiotherapy in bone metastases	90.38% (188/208)
Hypercalcemia of malignancy	1.92% (4/208)
Spinal cord and nerve root compression	5.29% (11/208)
SRE: skeletal related events.	

### 2.4 骨转移的治疗情况

骨转移的治疗是在全身治疗的基础上辅以双磷酸盐、局部放疗、对症止痛治疗及针对病理性骨折和脊髓压迫的姑息手术治疗。详见表 6。

**6:** NSCLC骨转移的治疗 Therapy of bone metastases in NSCLC

	Percent & Number	Response rate
Paregoric	36.96% (119/322)	73.11% (87/119)
Bisphosphonate	52.80% (170/322)	47.06% (80/170)
Radiotherapy	58.39% (188/322)	77.13% (145/188)
Surgery	2.80% (9/322)	77.78% (7/9)
Chemotherapy	89.13% (287/322)	46.34% (133/287)

### 2.5 生存情况

#### 2.5.1 生存情况

322例患者中，6例失访，失访率为1.86%，至末次随访日176例患者死亡，140例生存。发生骨转移后的1年生存率为40.99%，中位生存期为12.90个月。骨转移后无或SRE者的1年生存率分别44.75% *vs* 36.17%，中位生存期为14.74个月 *vs* 12.25个月(*χ*^2^=5.268, *P*=0.022)，二者有统计学差异。见图 1。

**1:**
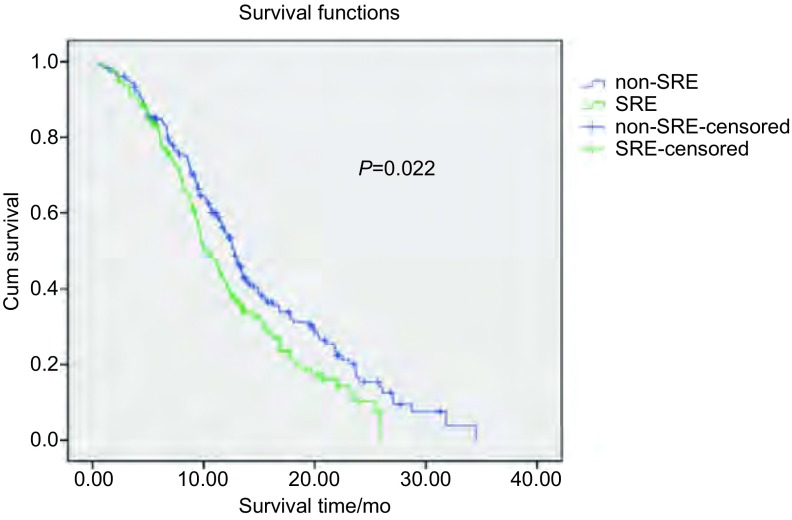
SRE与生存期的关系 Relationship of SRE and survival time

#### 2.5.2 单因素分析结果

病理类型、骨转移数量、是否合并其它部位转移、是否出现SRE、双磷酸盐治疗、局部姑息放射治疗、无症状骨转移、承重骨有无转移与预后相关。

#### 2.5.3 *Cox*多因素回归分析

患者病理诊断非腺癌(RR = 1.123, *P* = 0.018)，骨转移病灶数少于3个(RR = 1.366, *P* = 0.046)，单纯骨转移(RR = 1.497, *P*=0.008)为有益于生存期的预后因素(*P* < 0.05)。见表 7。

**7:** *Cox*回归变量表 *Cox* proportional hazards regression model multivariable analysis

	B	SE	Wald	df	Sig.	Exp(B)	95%CI Exp(B)	
							Lower	Upper
Pathology	0.116	0.049	5.609	1	0.018	1.123	1.020	1.236
Radiotherapy	0.072	0.158	0.207	1	0.649	1.075	0.788	1.465
Bisphosphonate	-0.018	0.144	0.015	1	0.902	0.982	0.741	1.303
SRE	0.407	0.256	2.528	1	0.112	1.502	0.910	2.481
Syndrome	-0.007	0.092	0.006	1	0.937	0.993	0.830	1.188
Mono bone metastases	0.403	0.152	7.052	1	0.008	1.497	1.111	2.015
Bearing bone	0.211	0.143	2.169	1	0.141	1.235	0.933	1.634
Numbers of bone metastases	0.312	0.156	3.988	1	0.046	1.366	1.006	1.854

## 3 讨论

骨转移的发生率与原发癌的病理类型有关。本组观察到骨转移患者中病理类型是腺癌的最多，为52.17% (168/322)，其次是鳞癌32.30%(104/322)。肺癌易发生骨转移的原因是：肺循环的血流丰富，脱落的肿瘤细胞易随血流进入肺循环和体循环到达全身骨骼系统；椎体、骨盆及肋骨等富含具有造血功能的红色骨髓，血流丰富，且毛细血管丰富，癌细胞易于定植而发生转移。肺腺癌多发生于肺边缘的杯状细胞或黏液细胞，向管外生长，易发生早期转移及侵犯血管淋巴管，继而发生全身转移；同时肺腺癌易出现局部侵犯累及肋骨及胸椎，是本组患者腺癌发生骨转移多的主要原因^[3]^。根据骨转移发生率高低排序，骨转移好发部位如下：脊柱、骨盆、股骨、肋骨、胸骨，与文献^[4-7]^报道相似。

《肺癌骨转移诊疗专家共识(2014版)》 ^[4]^将ECT作为初筛手段。因其为非特异性的骨骼显像方法，灵敏度高，但特异性低，尤其是孤立性病灶，需排除骨代谢疾病及陈旧性骨病。本组ECT示1个-2个浓聚灶者和大于或等于3个浓聚灶者，经CT/MRI证实率分别为：50.79%(32/63)和80.56%(203/252)。经*Pearson*卡方检验，两组有统计学差异(*χ*^2^=23.562, *P* < 0.001)。通过本组数据分析显示ECT敏感性为97.82%，特异性为54.32%，准确性为77.66%，漏诊率为22.33%，假阳性率为28.73%，假阴性率为4.43%。与高等^[8]^报道的结果一致。提示在ECT显示大于或等于3个以上浓聚灶者确诊骨转移的几率远远高于ECT显示2个以下浓聚灶的患者。因此对于ECT显示为1个-2个病灶者，进一步行CT/MRI等检查，并结合临床做出骨转移诊断尤为重要，以减少假阳性。本组7例ECT阴性患者，因有临床症状而行影像学检查证实骨转移，提示ECT作为骨转移的初筛手段，虽有较高的灵敏性，但亦有假阴性可能。此时骨转移病灶可能为溶骨性破坏，故不能通过反映骨盐代谢程度的ECT检测得到阳性诊断。因此临床上对具有固定疼痛部位的患者，尽管ECT阴性，亦应考虑对相应部位进行CT/MRI/X线，甚至PET-CT检查，以减少ECT的假阴性发生率。

NSCLC骨转移患者发生SRE将极大的降低生活质量，缩短生存期。Tsuya等^[7]^报道：SRE明显缩短肺癌骨转移患者生存时间。本回顾性研究显示无SRE的患者生存期长于发生SRE者[1年生存率分别为44.75%(nonSRE) *vs* 36.17%(SRE)；中位生存期分别为14.74个月 *vs* 12.25个月(*P*=0.022)]，双磷酸盐治疗可以改善生活质量，减少SRE发生，延长生存期^[6, 9]^。*Cox*回归分析显示：病理组织学非腺癌、骨转移病灶数少于3个、单纯骨转移是NSCLC骨转移的独立预后因素。无SRE发生者，Exp(B)=1.502，*P*=0.112。因统计设计中*P* < 0.05为入选标准，因此未成为独立预后因素。已呈现成为独立预后因素的趋势，尚需增加病例资料，进一步证实。

近年来，通过对骨标志物的研究发现，多种实体瘤骨转移治疗前骨标志物的升高，与SRE发生率升高，生存期降低有关，可以作为预测疗效的标志之一；根据骨标志物的水平进行个体化治疗的临床实验正在进行中。这些骨标志物包括：尿NTX、血清BALP、LDH和OPN(Osteopontin)等^[10, 11]^。NTX升高与SRE发生的风险呈正相关，NTX升高者骨转移进展和死亡风险是正常者的2倍，而且使用唑来磷酸可以降低35%的危险度^[9]^。81%的NTX升高的乳腺癌患者，使用唑来磷酸后降为正常；血清LDH的升高亦是乳腺癌骨转移进展和生存的重要预后指标，基线血清ALP小于正常值高限及经唑来磷酸治疗后ALP降至正常值高限以下者预后较好^[12, 13]^。大部分骨转移患者化疗及双磷酸盐治疗前后，LDH和ALP发生改变，提示此两项标志物可能反映骨转移治疗疗效，值得进一步探讨。本组患者未行相关检测。双磷酸盐治疗可以有效抑制破骨细胞活性，控制溶骨性骨破坏从而达到止痛，减少SRE的发生的作用^[14-16]^。

综上所述，本研究通过大样本回顾性分析显示肺腺癌骨转移发生率高，常见转移部位为脊柱、骨盆。ECT异常浓聚灶数目与转移确诊有关，出现SRE的患者生存期短于未发生SRE者。*Cox*回归分析显示：病理组织学非腺癌、骨转移病灶数少于3个、单纯骨转移是NSCLC骨转移的独立预后因素。
